# Down-Regulation of ABCA7 in Human Microglia, Astrocyte and THP-1 Cell Lines by Cholesterol Depletion, IL-1β and TNFα, or PMA

**DOI:** 10.3390/cells12172143

**Published:** 2023-08-25

**Authors:** Joel P. Wiener, Sindy Desire, Viktor Garliyev, Nicholas Lyssenko III, Domenico Praticò, Nicholas N. Lyssenko

**Affiliations:** Alzheimer’s Center at Temple, Department of Neural Sciences, Lewis Katz School of Medicine, Temple University, Philadelphia, PA 19140, USA

**Keywords:** ABC transporter, Alzheimer’s disease, cholesterol metabolism, cytokines, microglia, astrocytes, THP-1 cells, cyclodextrin, rosuvastatin, inflammation

## Abstract

Adenosine triphosphate-binding cassette transporter subfamily A member 7 (ABCA7) is a major risk factor for Alzheimer’s disease. Human neural cell lines were used to investigate the regulation of ABCA7 expression by cholesterol and pro-inflammatory cytokines. Cholesterol was depleted by methyl-β-cyclodextrin, followed by treatment with rosuvastatin to suppress de novo synthesis, while the cells underwent adjustment to low cholesterol. Cholesterol depletion by 50–76% decreased ABCA7 expression by ~40% in C20 microglia and ~21% in A172 astrocytes but had no effect on the protein in SK-N-SH neurons. Cholesterol depletion also suppressed ABCA7 in HMC3 microglia. Previously, cholesterol loss was reported to up-regulate ABCA7 in murine macrophages. ABCA7 was down-regulated during PMA-induced differentiation of human THP-1 monocytes to macrophages. But, cholesterol depletion in THP-1 macrophages by ~71% had no effect on ABCA7. IL-1β and TNFα reduced ABCA7 expression in C20 and HMC3 microglia but not in A172 astrocytes or SK-N-SH neurons. IL-6 did not affect ABCA7 in the neural cells. These findings suggest that ABCA7 is active in regular homeostasis in human neural cells, is regulated by cholesterol in a cell type-dependent manner, i.e., cholesterol depletion down-regulates it in human neuroglia but not neurons, and is incompatible with IL-1β and TNFα inflammatory responses in human microglia.

## 1. Introduction

Adenosine triphosphate-binding cassette transporter subfamily A member 7 (ABCA7) has abundant evidence for its involvement in Alzheimer’s disease (AD) pathogenesis from human genomic and genetic investigations, AD animal models, functional brain imaging in live individuals and postmortem studies of the human brain [1,2,3,4,5]. Nonetheless, many aspects of ABCA7 function and the mode of its involvement with the disease remain obscure. In particular, two critical but incompletely answered questions concern the role of ABCA7 in cholesterol metabolism and inflammation.

Like ABCA1, a closely related member of subfamily A, ABCA7 mediates formation of lipoprotein that consists of phospholipid, cholesterol and exchangeable apolipoprotein (such as apolipoprotein A-I and E) [6,7]. Because lipid for lipoprotein assembly comes from intracellular sources, lipoprotein formation entails phospholipid and cholesterol removal from the cell. However, while ABCA1 decreases in expression after intracellular cholesterol depletion, as would be expected for a protein that drives cell cholesterol efflux, ABCA7 has been reported to increase in expression via sterol regulatory element-binding protein 2 (SREBP-2) in cholesterol-depleted murine fibroblasts and macrophages [8,9]. We recently showed that ABCA7-lipoprotein contains less cholesterol than ABCA1-lipoprotein, likely because of lower cholesterol solubility in the phospholipid bilayer of ABCA7-lipoprotein than ABCA1-lipoprotein [10]. While common variants at the loci of *ABCA1* and *ABCA8*, another structurally related lipid transporter [11], are associated with plasma cholesterol measures, this is not the case for the variants at the *ABCA7* locus, in human populations [12,13,14,15]. Furthermore, disruption of intracellular cholesterol metabolism [16,17], cholesterol depletion [18,19,20] or up-regulation of SREBP-2 [21] affect expression of the genes for ABCA1 and ABCA8 but not ABCA7 in many tissues. Cumulatively, the present evidence lends itself to the interpretation that ABCA7 is associated with cholesterol metabolism in a more delimited and contextual manner than ABCA1 and ABCA8.

Reduction in *Abca7* expression has been reported to attenuate efferocytosis, a step in inflammation resolution, in murine macrophages and non-professional phagocytes [9,22,23]. This and the above finding about the increase in *Abca7* in cholesterol-depleted macrophages have led to the proposal that ABCA7 links cholesterol metabolism and inflammatory responses in phagocytic cells, i.e., low cholesterol levels induce an elevation in the expression of ABCA7, which in turn promotes macrophage phagocytic activity [24]. It should be noted that most of the studies into the role of ABCA7 in cholesterol metabolism and inflammation have been conducted in mouse models. Here, we show that cholesterol depletion reduces ABCA7 levels in human microglia and astrocyte but not neuronal cells; differentiation of human THP-1 monocytes to macrophages using phorbol 12-myristate 13-acetate (PMA) is associated with a decrease in ABCA7, but then subsequent cholesterol depletion in THP-1 macrophages has no effect on ABCA7 levels; while interleukin 1β (IL-1β) and tumor necrosis factor α (TNFα) suppress ABCA7 levels in human microglia but not astrocyte or neuronal cells. These findings indicate that the mode of ABCA7 regulation vis-à-vis cholesterol metabolism in human neuroglia and monocyte-derived macrophages is not the same as in mouse macrophages and that ABCA7 functions are incompatible with IL-1β and TNFα inflammatory responses.

## 2. Materials and Methods

### 2.1. Cell Lines and Culture

The following cell lines and media for routine growth of these cell lines were used: C20 (a kind gift from Dr. Alvarez-Carbonell [25])—DMEM with 4.5 mg/mL glucose, no pyruvate (Gibco, Thermo Fisher Scientific, Waltham, MA, USA), 5% fetal bovine serum (FBS), antibiotics; HMC3 and SK-N-SH (American Type Culture Collection [ATCC])—EMEM with 2 mM L-glutamine, 1 mM sodium pyruvate (ATCC), 10% FBS, and antibiotics; A172 and THP-1 (ATCC)—RPMI-1640 without L-glutamine (Gibco, Thermo Fisher Scientific, Waltham, MA, USA), 10% FBS, 2 mM L-alanyl-L-glutamine dipeptide (GlutaMAX; Gibco, Thermo Fisher Scientific, Waltham, MA, USA), antibiotics; BHK-ABCA1 (a kind gift from Dr. Chongren Tang [26]) and BHK-ABCA7 [10]—DMEM with 4.5 mg/mL glucose, no pyruvate (Gibco, Thermo Fisher Scientific, Waltham, MA, USA), 10% FBS, and antibiotics. All cells were grown at 37 °C in 5% CO_2_. For differentiation into macrophages, THP-1 cells were treated with 10 ng/mL PMA (P8139; Sigma-Aldrich, St. Louis, MO, USA) for 24 h.

### 2.2. Cell Cholesterol Depletion

Confluent cells were washed with FBS-free medium and treated with either 10 mM methyl-β-cyclodextrin (MβCD; M7439, Sigma-Aldrich, St. Louis, MO, USA) dissolved in FBS-free medium or FBS-free medium without MβCD for 45 min at 37 °C in 5% CO_2_. After that, the medium was removed, and the cells were washed with FBS-free medium twice. The cells that were treated with MβCD were incubated in FBS-free medium supplemented with 5 μM rosuvastatin (SML1264, Sigma-Aldrich, St. Louis, MO, USA) for 24 h, while the cells that were treated with medium without MβCD were incubated with the same FBS-free medium supplemented with 0.35% DMSO (Sigma-Aldrich, St. Louis, MO, USA) for the same duration. In the initial cholesterol depletion assays, cells were not treated with MβCD and were incubated with 5 μM rosuvastatin in FBS-free medium for 24 h.

### 2.3. Modified Amplex Red Assay for Measuring Cell Cholesterol

Intracellular cholesterol concentrations were measured using a modified protocol for the Amplex Red Cholesterol Assay Kit (A12216, Thermo Fisher Scientific, Waltham, MA, USA), as described in [27]. Cell cholesterol was extracted with hexane:isopropanol (3:2, *v*/*v*) for 10 min with gentle shaking. The extracts and plates with the extracted cells were placed into a fume hood to evaporate residual solvent. The cells were then lysed by incubation in 0.2 M NaOH at 37 °C for 3 h, followed by agitation on a shaker at room temperature for 5 min. The lysates were collected, stored at −20 °C, and measured for protein concentration using a Pierce BCA Protein Assay Kit (Thermo Fisher Scientific, Waltham, MA, USA). Cholesterol standards (0.31–20 μg/mL) were prepared using the cholesterol solution provided with the Amplex Red Kit using a 1-to-100 dilution for the highest standard, followed by a serial 1-to-2 dilution starting from the highest standard in isopropanol:NP40 (9:1, *v*/*v*). Isopropanol:NP40 was used as a blank. After solvent evaporation, the extracts were dissolved in 1 mL/tube of isopropanol:NP-40 by incubating at room temperature for 30 min and vortexing. Aliquots of the samples, standards, and blank (40 μL each) were combined with 10 μL/well of 100 U/mL catalase (from bovine liver; C30, MilliporeSigma, Burlington, MA, USA) in 96-well plates and incubated at 37 °C for 15 min. Then, 150 μL/well aliquots of the 1X reaction buffer were added and mixed in using a multichannel pipetter, and the plates were incubated at 37 °C for an additional 15 min and then read in a SpectraMax i3x multi-mode microplate reader (excitation 530 nm/emission 580 nm; Molecular Devices, San Jose, CA, USA). The composition of the 1X reaction buffer was as the following: 1.08 mL 5X reaction buffer, 1.08 mL 1 M NaCl, 36 μL 200 U/mL horseradish peroxidase, 19 μL 200 U/mL cholesterol esterase, 9 μL 200 U/mL cholesterol oxidase, 3.076 mL deionized water and 100 μL 20 mM Amplex Red (10-acetyl-3,7-dihydroxyphenoxazine) solution. NaCl and Amplex Red were dissolved in water and DMSO, respectively, and the other reagents were dissolved in 1X reaction buffer. The composition of the 5X reaction buffer was as the following: 20 mL 0.5 M potassium phosphate, pH 7.4, 0.25 M NaCl, 25 mM cholic acid and 0.5% Triton X-100. All enzymes and Amplex Red were from the kit; *Pseudomonas* sp. esterase was from Sigma-Aldrich (C1403, St. Louis, MO, USA). Cholesterol concentrations were expressed in μg of cholesterol per mg of cell protein.

### 2.4. Cell Treatment with Mifepristone, LXR Agonist and U18666A

BHK-ABCA1 and BHK-ABCA7 cells carry stably integrated human *ABCA1* and *ABCA7*, respectively, under the control of a mifepristone-inducible promoter from the GeneSwitch system (Thermo Fisher Scientific, Waltham, MA, USA). BHK-ABCA1 and BHK-ABCA7 cells were treated with 10 nM mifepristone (Invitrogen, Thermo Fisher Scientific, Waltham, MA, USA) in FBS-free medium for 24 h to induce *ABCA1* or *ABCA7* expression. Cells were treated with 2 μM T0901317 (575310, MilliporeSigma, Burlington, MA, USA) in FBS-free medium for 24 h to induce expression of liver X receptor (LXR)-regulated genes. U18666A was applied to cells at 7 μM together with 5 μM rosuvastatin.

### 2.5. Cell Treatment with Pro-Inflammatory Cytokines

Human IL-1β, IL-6 and TNFα were purchased from PeproTech (Cranbury, NJ, USA) and prepared using endotoxin-free, fraction V, fatty acid-poor bovine serum albumin (BSA, 0.1% *w*/*v* final concentration in 20 μg/mL stocks of the cytokines) and stored as recommended by the manufacturer. Confluent cells grown in 6-well plates were treated with 20 ng/mL IL-1β, IL-6, TNFα or 0.0001% BSA in FBS-free medium for 24 h. Cell medium and cell lysates were then collected for further analysis.

### 2.6. Western Immunoblotting

Cells grown in 6-well plates were washed with ice-cold DPBS and lysed by incubating in 50–70 μL/well RIPA buffer (Sigma-Aldrich, St. Louis, MO, USA) supplemented with a protease inhibitor cocktail at 1:100 dilution from the stock solution (104 mM AEBSF, 80 μM aprotinin, 4 mM bestatin, 1.4 mM E-64, 2 mM leupeptin, 1.5 mM pepstatin A; P8340, Sigma-Aldrich, St. Louis, MO, USA) and 20 µg/mL calpain inhibitor I (A6185, Sigma-Aldrich, St. Louis, MO, USA) on ice for 10 min. Lysates were collected by scraping and centrifuged at 14,000× *g* for 15 min at 4 °C. Protein concentration in the supernatants was measured using a Pierce BCA Protein Assay (Thermo Fisher Scientific, Waltham, MA, USA). Lysate aliquots were combined with sample buffer and reducing agent (Bio-Rad, Hercules, CA, USA), vortexed, pulse-centrifuged and either kept at room temperature for 15–30 min or heated in a 60 °C water bath for 10 min; the lysate samples heated at 60 °C were allowed to cool at room temperature for 5–10 min and pulse-centrifuged. (Note, heating lysates at >90 °C significantly reduces immunodetectable ABCA7, while heating lysates at ≥60 °C significantly reduces immunodetectable ABCA1; see Appendix A.) Lysate protein (15–30 μg total protein per lane) was resolved on 3–8% Criterion XT tris-acetate protein gels (26 wells per gel; 15 μL loading volume) (Bio-Rad, Hercules, CA, USA) in XT tricine running buffer at 150 V for 75 min. The protein was then transferred onto nitrocellulose membrane (Bio-Rad, Hercules, CA, USA) using Hoefer TE70XP semi-dry transfer units (80–82 mA for 1 h, Hoefer Inc., Holliston, MA, USA) or Trans-Blot SD semi-dry transfer cells (15–20 V for 1 h; Bio-Rad, Hercules, CA, USA). The membrane was treated with LI-COR Intercept (TBS) blocking buffer (LI-COR Biosciences, Lincoln, NE, USA) on a rocker at 10–15 rpm for 1 h and, in most instances, cut horizontally at the 50 kDa ladder marker. The upper portion was probed with one or more of the following antibodies: anti-ABCA7, anti-LDLR, anti-ABCA1 and anti-LRP1 antibody, while the lower portion was probed with an anti-GAPDH or anti-β-actin antibody. The following antibodies were used: mouse monoclonal anti-human ABCA7 (E11) (1:100 dilution; sc-377335, Santa Cruz Biotechnology, Dallas, TX, USA; this antibody was previously validated [5]), rabbit polyclonal anti-ABCA1 (1:500 dilution; NB400-105, Novus Biologicals, Centennial, CO, USA), rabbit monoclonal anti-LDLR (EP1553Y) (1:500 dilution; ab52818, Abcam, Cambridge, United Kingdom), rabbit monoclonal anti-LDLR (SJ0197) (1:1000 dilution; MA5-32075, Thermo Fisher Scientific, Waltham, MA, USA), rabbit monoclonal anti-LRP1 (EPR3724) (1:10,000 dilution; ab92544, Abcam, Cambridge, United Kingdom), rabbit monoclonal anti-GAPDH (1:500 dilution; 2118, Cell Signaling Technology, Danvers, MA, USA) and mouse monoclonal anti-β-actin (C4) (1:200 dilution; sc-47778, Santa Cruz Biotechnology, Dallas, TX, USA). The secondary antibodies were IRDye 800CW-conjugated goat anti-rabbit polyclonal (1:5000 dilution) and IRDye 800CW-conjugated donkey anti-mouse polyclonal (1:3000 dilution) (both from LI-COR Biosciences, Lincoln, NE, USA). Membranes were scanned with an Odyssey CLx imaging system (LI-COR Biosciences, Lincoln, NE, USA). Protein expression quantification was conducted using Image Studio version 5.2 or ImageJ version 1.54b. Areas encompassing the bands of interest were manually drawn using either the rectangle or freehand tool. In Image Studio, the band intensity was adjusted for the background, which was measured as the median of all the pixels in an area that was larger by one pixel on all sides than the selected area. In ImageJ, images were inverted and converted to 16-bit format before band quantification. The ratios of fluorescence intensity from the protein of interest bands to the GAPDH band were taken as normalized protein expression.

### 2.7. IL-1β and TNFα ELISA

TNFα and IL-1β were measured using, correspondingly, TNFα Human High Sensitivity ELISA (BMS223HS; Invitrogen, Thermo Fisher Scientific, Waltham, MA, USA) and IL-1β Human ProQuantum Immunoassay Kits (A35574; Invitrogen, Thermo Fisher Scientific, Waltham, MA, USA). Total protein content in cell lysates was quantified using BCA. Cell cytokine release was expressed in pg of cytokine/mg of cell protein per 24 h.

### 2.8. Extraction and Re-Analysis of Publicly Available Microarray and RNA Sequencing Data

Google Scholar and the Gene Expression Omnibus (GEO) were searched for studies and datasets of interest. Fold changes and unadjusted *p*-values (because a specific narrow hypothesis regarding each gene was tested) were either extracted from the summary tables or derived by data re-analysis using GEO2R with the default settings. When count measures were provided, the genes of interest counts were extracted, tested for normality and analyzed by *t*-test. A meta-analysis on *p*-values was conducted with the Fisher’s method using the poolr package for R [28] without adjustment for dependency.

### 2.9. Statistical Analysis and Figure Assembly

Data were assessed for normality by the Shapiro-Wilk test and then analyzed by either the paired or unpaired two-tailed *t*-test, or repeated measures or ordinary ANOVA with correction for multiple comparisons by controlling the false discovery rate at 0.05 using the two-stage step-up method of Benjamini, Krieger, and Yekutieli. The Greenhouse-Geisser correction was applied to the repeated measures ANOVA. Ratio-paired *t*-tests were used where noted. Estimation plots were drawn where paired *t*-tests were used. Estimation plots show the change in the variable for each pair of samples on the left side and the difference between the paired means, the mean of the differences, and confidence intervals on the left. Correlation plots were drawn, and Pearson’s *r* and *p* were calculated for ratio-paired *t*-tests to demonstrate the correlation in the variable for the paired samples. To calculate the change in the protein of interest on the percentage basis, the control mean was taken as 100%, and each test value was expressed as a percentage of the control mean. Data were analyzed and graphed with GraphPad Prism 9.5.0. The values indicated in the text and the error bars in all graphs are the mean ± standard deviation. Figures were assembled from Odyssey CLx images and GraphPad Prism graphs using Krita version 4.4.5 (Stichting Krita Foundation, Deventer, the Netherlands).

## 3. Results

### 3.1. Cholesterol Depletion Down-Regulates ABCA7 Expression in Human Microglia and Astrocyte but Not Neuronal Cells

**Figure 1:**
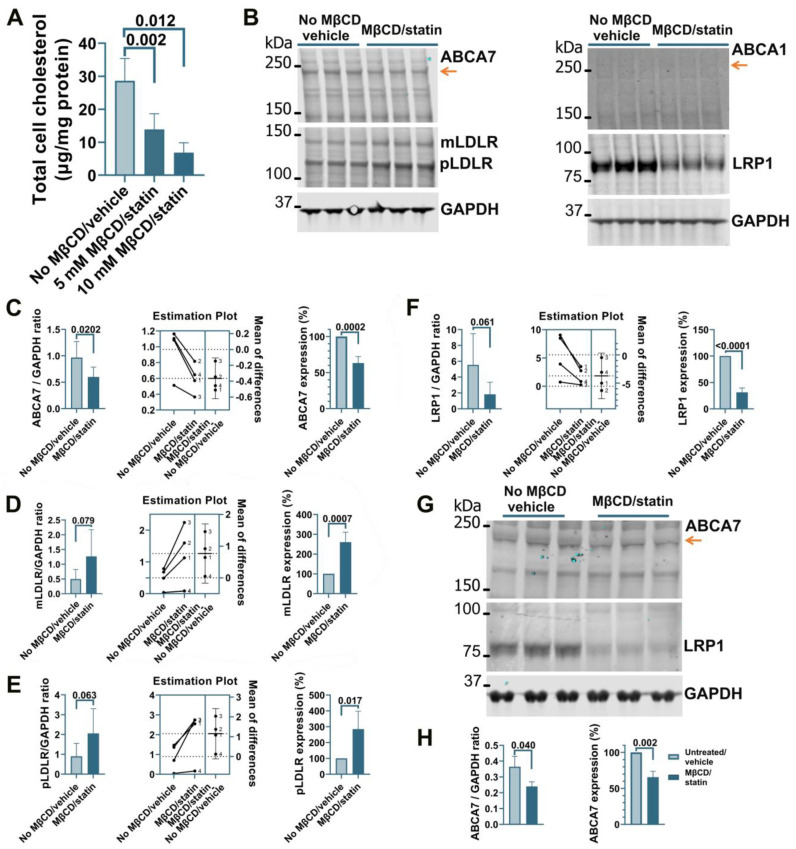
Reduced ABCA7 protein levels after cholesterol depletion in human C20 and HMC3 microglia cells. (**A**) C20 cells treated with the indicated concentrations of MβCD for 45 min and then kept in 5 μM rosuvastatin without FBS for 24 h had significantly lower cholesterol concentrations. The 10 mM MβCD/5 μM rosuvastatin treatment significantly reduced the levels of ABCA7 (**B**,**C**) and LRP1 (**B**,**F**), and significantly increased the levels of mLDLR (**B**,**D**) and pLDLR (**B**,**E**). (**B**) ABCA1 expression was at the limit of detection before and after the treatment. (**G**,**H**) HMC3 cells depleted in cholesterol using the 10 mM MβCD/5 μM rosuvastatin treatment had significantly lower levels of ABCA7 and LRP1 protein. Statistical analysis in (**A**)—ordinary ANOVA; (**C**–**F**)—paired *t*-test for the ratio values (the experiments are numbered in the estimation plots, n = 4) and unpaired *t*-test for the changes in the protein expression on the percentage basis with the average value in the controls set at 100%; (**H**)—*t*-test.

Treatment of human microglia cells C20 with 5 μM rosuvastatin for 24 h did not reduce intracellular cholesterol levels significantly (0.84 ± 9.90% reduction, n = 3). A modified procedure was implemented whereby cells were first exposed to MβCD to strip cholesterol and then kept in FBS-free medium supplemented with rosuvastatin for 24 h to prevent cholesterol replenishment through de novo synthesis while cell metabolism adjusted to low cholesterol levels. C20 cells treated with 5 mM MβCD/5 μM rosuvastatin or 10 mM MβCD/5 μM rosuvastatin had, correspondingly, 52 ± 16.7% and 76 ± 10.3% less cholesterol than control cells (Figure 1A). The higher 10 mM MβCD concentration and more severe cholesterol depletion were chosen for further work. Cholesterol depletion using the 10 mM MβCD/5 μM rosuvastatin treatment reduced ABCA7 protein levels in C20 cells by 36.8 ± 9.08% (*p* = 0.0002, n = 4; Figure 1B,C). Up-regulation of low-density lipoprotein receptor (LDLR) expression is a well-established common response to cholesterol depletion [29,30]. LDLR levels in the same C20 cells were assessed as a positive control. Amounts of the mature (mLDLR, the band migrating at the larger kDa size) and precursor (pLDLR, the band migrating at the smaller kDa size [30]) LDLR were significantly higher in MβCD/rosuvastatin-treated cells (Figure 1B,D,E). Low-density lipoprotein receptor 1 (LRP1) restrains cholesterol accumulation in the cell during cholesterol abundance but undergoes cleavage in the β chain (which is ~85 kDa in size) upon cholesterol depletion [31,32]. LRP1 β chain levels were quantified as a second positive control and were found to be significantly reduced in MβCD/rosuvastatin-treated cells (Figure 1B,F). An increase in LDLR and loss of LRP1 indicated that MβCD/rosuvastatin-treated cells indeed experienced low cholesterol levels. ABCA1 is up-regulated to remove excessive intracellular cholesterol but is otherwise expressed at a low basal level [33]. ABCA1 expression was also assessed to evaluate the state of cholesterol metabolism in C20 cells. The ABCA1 protein was at a barely detectable level in control and MβCD/rosuvastatin-treated cells, indicating that intracellular cholesterol was at a lower level than that required to up-regulate ABCA1 even before cholesterol depletion (Figure 1B). The effect of cholesterol depletion on ABCA7 expression was investigated in a second human microglia cell line, HMC3. HMC3 cells subjected to the same cholesterol depletion procedure as C20 cells had reduced ABCA7 (34.3 ± 8.16%) and LRP1 protein levels (Figure 1G,H). LDLR could not be detected in HMC3 cells with two different antibodies that specifically recognized this protein in C20 cells, likely because of defects in LDLR glycosylation and precursor degradation in this cell line [30]. Expression of ABCA1 in untreated HMC3 cells was also very low (see below, Figure 4C).

**Figure 2:**
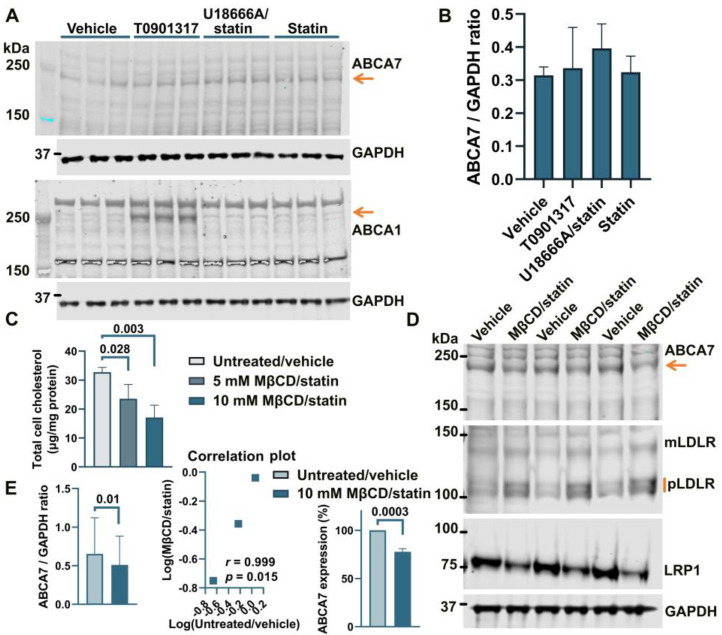
Reduction in the ABCA7 protein in response to cholesterol depletion in astrocyte A172 cells. (**A**,**B**) Inhibition of cholesterol synthesis with 5 μM rosuvastatin for 24 h in A172 cells reduced intracellular cholesterol by 16.2 ± 3.59% but did not translate into a reduction in ABCA7 protein levels. (The T0901317 and U18666A/statin treatment lanes are shown to avoid splicing the gel picture; these results are discussed in Section 3.2). (**C**–**E**) The 10 mM MβCD/5 μM rosuvastatin treatment was used to bring about greater decreases in intracellular cholesterol that led to moderate but significant reductions in the ABCA7 protein. Statistical analysis in (**B**)—repeated measures ANOVA (F = 1.27, *p* = 0.38, n = 3); (**C**)—ordinary ANOVA; (**E**)—ratio-paired *t*-test and Pearson’s *r* and *p* for the effectiveness of pairing for the ratio values and unpaired *t*-test for the changes in the protein expression on the percentage basis with the average value in the controls set at 100%.

Treatment of human astrocyte cells A172 with 5 μM rosuvastatin for 24 h caused a moderate but consistent reduction of 16.2 ± 3.59% (*p* = 0.0001, n = 4) in intracellular cholesterol. However, this moderate downshift in cholesterol content had no effect on ABCA7 protein levels (F = 1.27, *p* = 0.38, n = 3; Figure 2A,B). The two-step MβCD/rosuvastatin procedure was applied to achieve greater cholesterol depletion. A172 cells treated with 5 mM MβCD/5 μM rosuvastatin or 10 mM MβCD/5 μM rosuvastatin had, correspondingly, 28.0 ± 15.04% and 47.8 ± 13.07% less cholesterol than control cells (Figure 2C). The 10 mM MβCD concentration and more severe cholesterol depletion were again chosen for subsequent assays. A172 cells treated with 10 mM MβCD/5 μM rosuvastatin had reduced levels of the ABCA7 protein by 21.4 ± 3.34% (*p* = 0.0004, n = 3) in comparison with control cells (Figure 2D,E). Cholesterol-depleted cells also exhibited higher expression of mLDLR and pLDLR and lower expression of LRP1 (Figure 2D). ABCA1 expression in A172 cells was at a minimal level before cholesterol depletion and after treatment with rosuvastatin alone (Figure 2A).

**Figure 3:**
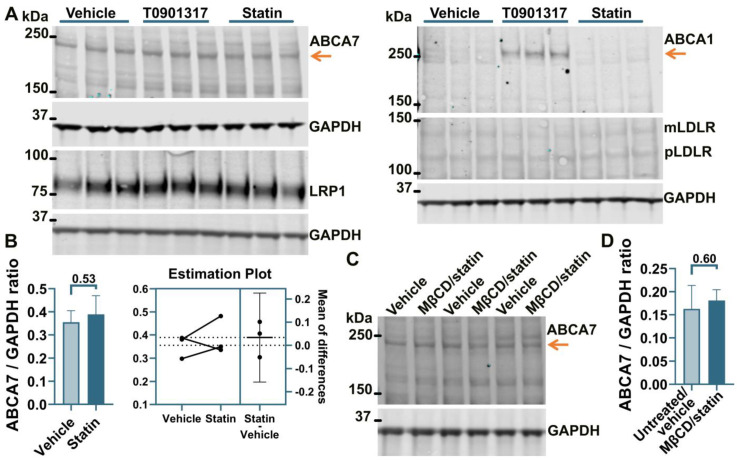
Cholesterol depletion did not affect ABCA7 in human neuronal SK-N-SH cells. (**A**) SK-N-SH cells were treated with the indicated compounds for 24 h, and then the level of the indicated proteins was assessed by Western. (**B**) There was no change in the ABCA7 protein level after a 24 h treatment with 5 μM rosuvastatin that reduced intracellular cholesterol in SK-N-SH cells by 49.4 ± 12.3%. (**C**,**D**) There was no change in ABCA7 levels after the 10 mM MβCD/5 μM rosuvastatin treatment. Statistical analysis in (**B**)—paired *t*-test; (**D**)—unpaired *t*-test.

Incubation of human neuronal cells SK-N-SH in 5 μM rosuvastatin for 24 h reduced intracellular cholesterol by 49.4 ± 12.3%. However, this reduction had no effect on the levels of ABCA7, mLDLR, pLDLR, or LRP1 (Figure 3A,B). ABCA1 expression was very low before and after rosuvastatin application (Figure 3A). The 10 mM MβCD/5 μM rosuvastatin treatment also did not affect ABCA7 levels (Figure 3C,D). Thus, cholesterol depletion caused a reduction in the protein level of ABCA7 in human microglia and astrocyte cells but had no consequence for ABCA7 expression in a human neuronal cell line.

### 3.2. No Effect of an LXR Agonist or the U18666A/Statin Co-Treatment on ABCA7 Expression in Human Neural Cell Lines

**Figure 4:**
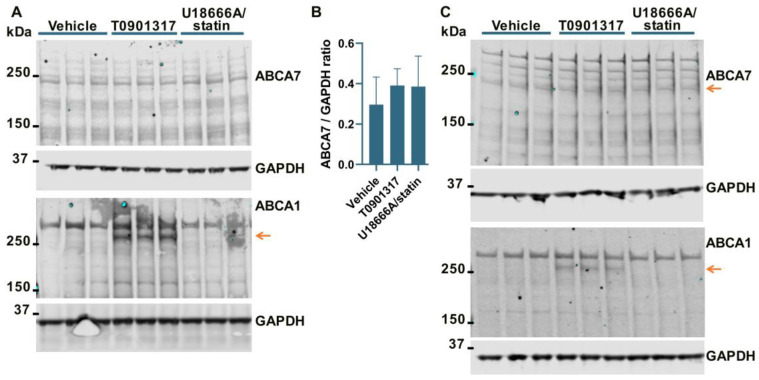
The LXR agonist T0901317 and U18666A/statin co-treatment did not affect the ABCA7 protein in human microglia cells. (**A**,**B**) The experiment with C20 cells was repeated four times, and the results were analyzed using repeated measures ANOVA without finding any significant difference (F = 1.85, *p* = 0.26). (**C**) T0901317 and the U18666A/statin co-treatment did not affect ABCA7 in HMC3 cells. As expected, T0901317 induced strong expression of ABCA1 in both cell types. The anti-ABCA1 antibody in one batch detected a spurious band at a higher molecular weight than ABCA1 ((**A**,**C**) and Figure 2A). The identity of the ABCA1 protein band was confirmed using BHK-ABCA1 cells. Subsequent batches of this same anti-ABCA1 antibody did not recognize the spurious band.

It has been previously shown that ABCA7 is not regulated by LXR in murine primary macrophages [34]. However, given the present results that human neural cells differ from murine macrophages with respect to the regulation of the ABCA7 protein by cholesterol depletion (i.e., cholesterol depletion down-regulates ABCA7 in human microglia and astrocyte cells and has no effect in a human neuronal cell line but up-regulates it in murine macrophages), it could be that neural cells are also different from macrophages with respect to ABCA7 regulation by LXR. An LXR agonist T0901317 was used to induce LXR activity. LXR strongly up-regulates ABCA1 expression [33]. ABCA1 levels were assessed as a positive control. Also, dual treatment with U18666A, a lysosomotropic compound that inhibits cholesterol transport out of lysosomes [35], and rosuvastatin was employed as a model of disrupted intracellular cholesterol metabolism. T0901317 strongly up-regulated ABCA1, but neither T0901317 nor U18666A/statin had an effect on ABCA7 expression in human microglia C20 (F = 1.85, *p* = 0.26, n = 4, Figure 4A,B) and HMC3 (Figure 4C) cells, in astrocyte A172 cells (n = 3; Figure 2A,B) or in SK-N-SH neuronal cells (Figure 3A).

### 3.3. PMA Treatment to Differentiate THP-1 Monocytes to Macrophages Induces a Reduction in ABCA7 Levels, but Subsequent Cholesterol Depletion in THP-1 Macrophages Has No Effect on ABCA7 Expression

**Figure 5:**
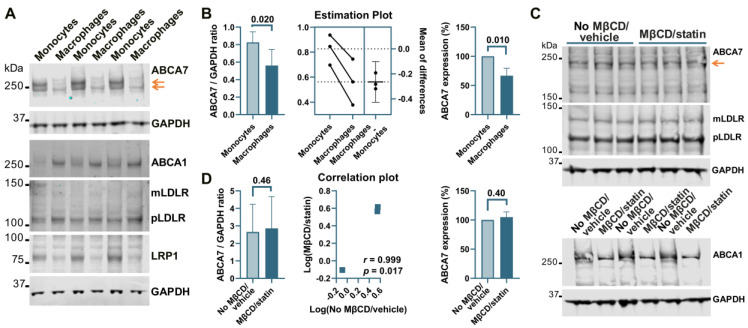
Regulation of ABCA7 levels in THP-1 cells. (**A**) PMA-mediated differentiation of THP-1 monocytes to macrophages induced down-regulation of ABCA7 and LRP1 and up-regulation of ABCA1, mLDLR, and pLDLR. (**B**) The reduction in ABCA7 in THP-1 macrophages relative to THP-1 monocytes was significant. (**C**,**D**) Depletion of cholesterol in THP-1 macrophages did not affect ABCA7 and LDLR levels but notably decreased expression of ABCA1. Statistical analysis in (**B**)—paired *t*-test for the ratio values and unpaired *t*-test for the percentage change in expression; (**D**)—ratio-paired *t*-test and Pearson’s *r* and *p* for the effectiveness of pairing for the ratio values and unpaired *t*-test for the percentage change in expression.

THP-1 cells, a widely used model of human monocytes and monocyte-derived macrophages, were investigated to determine whether the paradigm of ABCA7 regulation by cholesterol that has been discovered in studies with mouse tissue [8,9] also applies to human tissue. Human THP-1 monocytes were incubated with PMA to induce differentiation to macrophages. THP-1 monocytes strongly expressed two isoforms of ABCA7, one that migrated below the 250 kDa marker and corresponded to the product of the GENCODE transcript ENST00000263094.11 (NCBI RefSeq gene NM_019112.4) in size (predicted molecular weight 234.4 kDa) and another one that migrated above the 250 kDa marker (Figure 5A). Strong expression of the second isoform has been previously shown in certain cell types (see Appendix B). The <250 kDa isoform is by far the predominant isoform in C20, HMC3, A172, and SK-N-SH cells (Figure 1, Figure 2, Figure 3 and Figure 4) and, as we have previously shown [5], human whole-brain lysates. In comparison with THP-1 monocytes, THP-1 macrophages expressed very little of the >250 kDa isoform and 33.2 ± 12.7% (*p* = 0.01, n = 3) less of the <250 kDa isoform (Figure 5A,B). Furthermore, THP-1 macrophages had markedly increased levels of ABCA1, moderately elevated levels of mLDLR and pLDLR, and notably decreased levels of LRP1 (Figure 5A). Treatment of THP-1 macrophages with 10 mM MβCD/5 μM rosuvastatin decreased intracellular cholesterol by 71.2 ± 5.46% but had no effect on ABCA7 (*p* = 0.40, n = 3) and LDLR expression and markedly down-regulated ABCA1 (Figure 5C,D).

### 3.4. Suppression of the ABCA7 Protein by IL-1β and TNFα in Microglia but Not Astrocyte or Neuronal Cells

**Figure 6:**
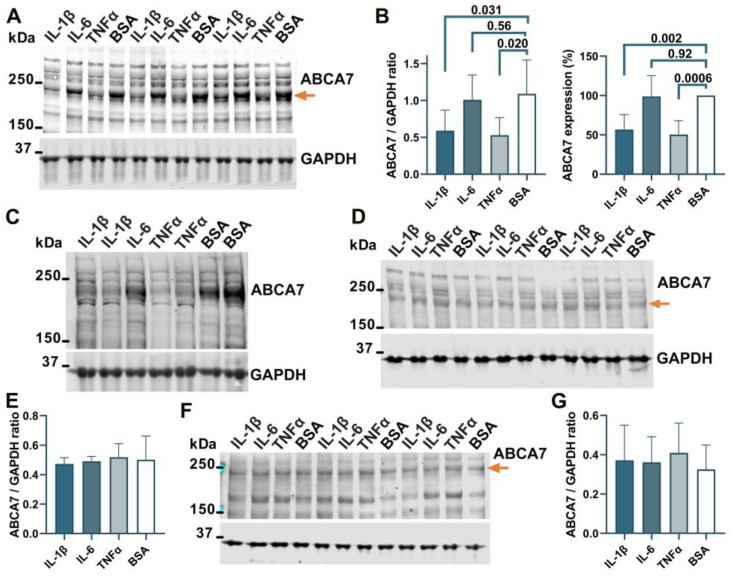
IL-1β and TNFα suppress ABCA7 in microglia C20 and HMC3 cells but not astrocyte A172 or neuronal SK-N-SH cells. (**A**,**B**) IL-1β and TNFα significantly reduced ABCA7, while IL-6 had no effect in C20 cells. (**C**) The same two cytokines markedly reduced ABCA7 in HMC3 cells. (**D**,**E**) None of the cytokines affected ABCA7 in A172 cells. (**F**,**G**) The cytokines also did not affect ABCA7 in SK-N-SH cells. Statistical analysis in (**B**)—repeated measures ANOVA (left panel) and ordinary ANOVA (right panel); (**E**)—ordinary ANOVA (F = 0.12, *p* = 0.94); (**G**)—repeated measures ANOVA (F = 1.2, *p* = 0.39).

**Figure 7:**
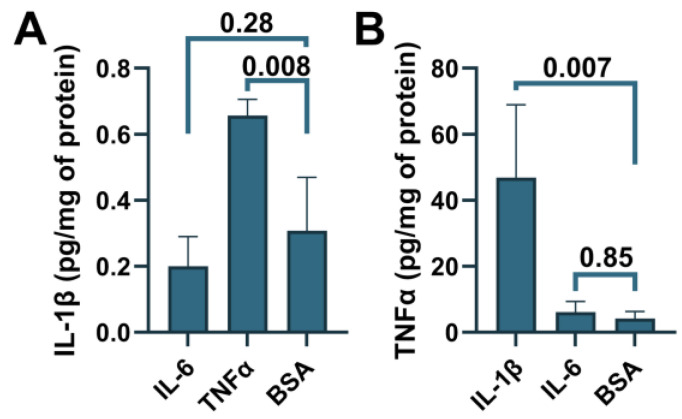
Induction of IL-1β secretion by TNFα and TNFα secretion by IL-1β in C20 cells. C20 cells were treated with the indicated cytokines, and then IL-1β (**A**) or TNFα (**B**) was measured in the cell medium using ELISA. Statistical analysis—ordinary ANOVA (n = 3).

To determine whether ABCA7 participates in inflammatory responses, C20, HMC3, A172 and SK-N-SH cells were treated with three major pro-inflammatory cytokines that are expressed by neuroglia [36], IL-1β, IL-6, or TNFα, followed by quantification of ABCA7 expression. IL-1β and TNFα inhibited ABCA7 levels by, correspondingly, 43.3 ± 19.87% and 49.6 ± 8.28% (*p* = 0.002 and 0.0006, n = 5) in C20 cells (Figure 6A,B) and also markedly suppressed ABCA7 expression in HMC3 cells (Figure 6C). IL-6 did not affect the transporter in C20 or HMC3 cells (Figure 6A–C), while none of the three cytokines affected ABCA7 in astrocyte A172 or neuronal SK-N-SH cells (Figure 6D–G). In cultured human primary microglia, IL-1β and TNFα are constitutively secreted in small amounts, modestly increase each other’s release, and are not stimulated by IL-6 [37,38,39]. To show that this regulatory pattern holds in the C20 cell line, C20 cells were exposed to IL-1β, IL-6 or TNFα, and IL-1β was measured in the medium from the cells exposed to IL-6 or TNFα, while TNFα was measured in the medium from the cells exposed to IL-1β or IL-6. Both IL-1β and TNFα were present in the medium from the carrier-treated cells (Figure 7). TNFα had a small (~2 fold) but significant positive effect on secretion of IL-1β (Figure 7A), as has been previously reported [25]. In contrast, IL-1β robustly (~11 fold) increased release of TNFα; IL-6 had no effect on IL-1β or TNFα secretion (Figure 7A,B).

## 4. Discussion

Cells within each tissue can be functionally classified as immune, supportive or primary [40,41]. In the central nervous system, the three classes are represented by, correspondingly, microglia, astrocytes and neurons. Each cell class participates in inflammation. However, while immune cells undergo dramatic changes in metabolism and overall homeostasis to mount an effective defensive response specific to a particular type of threat [42], supportive and primary cells up- or down-regulate select core processes to facilitate this response [40,41]. Consequently, regular physiological processes and protein activities fall into two categories, those that are compatible with inflammatory responses and therefore remain unaltered or are boosted during the inflammation proper and those that are incompatible with inflammatory responses and are therefore suppressed during the inflammation proper [40,41]. The intracellular free cholesterol concentration is strictly maintained at a set point in regular homeostasis but undergoes major shifts during the transition from regular metabolism to immunometabolism depending on the type of inflammatory response [42,43]. In macrophages in particular, signaling through the TNF receptor and Toll-like receptor (TLR)/myeloid differentiation primary response protein 88 (MyD88) pathways activates cholesterol synthesis, raises its uptake from the extracellular sources and increases intracellular cholesterol levels, while signaling through the interferon receptor has the opposite effect and reduces cholesterol synthesis, uptake and intracellular concentration [21,43,44,45]. Furthermore, exogenously-induced shifts in intracellular cholesterol can activate pro-inflammatory factors [46]. The purpose of the present work was to begin understanding how the ABCA7 functions fit in with regular and inflammatory homeostases and cholesterol metabolism in neural cells.

The present work makes the following two key findings. First, depletion of intracellular cholesterol suppresses ABCA7 in human microglia and astrocyte cell lines, i.e., immune and supportive, respectively, cell types, but does not affect it in a neuronal cell line, i.e., a primary cell type. In the astrocyte cell line, cholesterol depletion must be robust enough to reduce its level by ~50% in order to bring ABCA7 expression down, as a mild depletion that lowered the concentration by ~16% had no effect on the transporter. The differences between the neural cell types in the regulation of ABCA7 by cholesterol likely stem from the well-documented distinct roles each of these cell types performs in the overall brain cholesterol metabolism [47]. This finding that low cholesterol suppresses ABCA7 contrasts with the previously reported observations that cholesterol depletion induces an increase in ABCA7 expression in murine macrophages [8,9]. To determine whether this difference in the findings had come about owing to the type (microglia/astrocytes vs. macrophages) or the species (human vs. mouse) of the cells employed in those and our studies, THP-1 macrophages were investigated. ABCA7 remained unchanged, while ABCA1 was strongly down-regulated, by a very robust cholesterol depletion in this human macrophage model. This suggests existence of major species-specific divergences in the ABCA7 regulation by cholesterol. To determine how the present finding compares with other previous results, *ABCA7*/*Abca7* levels were assessed in six public gene expression datasets from the studies that investigated the effect of cholesterol lowering on unrelated to ABCA7 processes (Appendix C). Cholesterol depletion in those studies was deemed significant if *LDLR*/*Ldlr* expression was significantly increased and *ABCA1*/*Abca1* expression was significantly decreased. Cholesterol loss as assessed by the changes in *LDLR*/*Ldlr* and *ABCA1*/*Abca1* did not affect *ABCA7*/*Abca7* expression in human aortic smooth muscle cells, mouse liver, mouse cortical neural stem progenitor cells, human peripheral blood mononuclear cells or human monocytes; ABCA7 expression was, however, increased in human myoblasts. Cumulatively, our findings and the findings from refs. [8,9] and the public datasets suggest that ABCA7 does not react to changes in cholesterol concentrations in most cell types but when a response takes places it can be up- or down-regulation depending on the cell type or potentially cell species.

Second, ABCA7 functions are part of regular homeostasis and are incompatible with TNFα- and IL-1β-mediated inflammatory responses in neural immune cells but are compatible with these responses in neural supportive and primary cells. To buttress this finding in our work, *ABCA7*/*Abca7* levels were assessed in public gene expression datasets from the studies that investigated neuroglia phenotypes induced by IL-1β or TNFα for unrelated to ABCA7 purposes (Appendix D). *Abca7* was significantly down-regulated by TNFα in mouse microglia [48]. But meta-analyses of six datasets showed that *ABCA7*/*Abca7* was not suppressed by IL-1β or TNFα in human or mouse astrocytes. It should be noted that PMA-mediated differentiation of THP-1 monocytes to macrophages involves activation of an inflammatory response (i.e., NLRP3 priming; [49] and Wiener, unpublished observations). Down-regulation of ABCA7 in THP-1 macrophages relative to THP-1 monocytes suggests that ABCA7 functions are also not compatible with the PMA-induced inflammation phenotype. It was recently reported that *ABCA7* is down-regulated by a co-treatment of TNFα and interferon γ (IFNγ) in a mixed culture of human cortical astrocytes [50]. IFNγ alone suppresses ABCA7 in A172 astrocyte cells (Wiener, unpublished observations). Given these instances of the suppressive effect of inflammatory signaling on ABCA7, it may be that ABCA7 functions are incompatible with many inflammatory responses. Because there is a discord among the effects of cholesterol depletion, IL-1β, TNFα and IFNγ on ABCA7 expression (i.e., cholesterol depletion down-regulates ABCA7, while IL-1β and TNFα do not affect it, in the astrocyte cell line; furthermore, TNFα, which has been reported to increase intracellular cholesterol, and IFNγ, which has been reported to decrease intracellular cholesterol, both down-regulate ABCA7), it is likely that cholesterol and inflammation regulate ABCA7 independently.

The present findings chime well with the lipidostasis hypothesis of ABCA7 activity, which we have previously advanced [5]. The hypothesis contends that ABCA7 performs a main metabolic function by removing from the cell a lipid that accumulates during normal homeostasis of neural tissue and can cause neurodegeneration if not eliminated. A recent study suggests that prolonged high-demand cognitive work increases the brain concentration of glutamate, which then impedes cognition, i.e., normal functioning of the brain generates a metabolite that interferes with its further normal activity [51]. This scenario is the same as in the lipidostasis hypothesis, except the offending metabolite is a lipid. The present work shows that ABCA7 is part of normal physiology in three neural cell types. Another recent study reported that *Abca7* haplodeficient mice exhibit lower baseline levels of pro-inflammatory cytokines and mount a sluggish immune response when challenged with lipopolysaccharide [52]. Taking into account the latter study, our present findings and the observations that ABCA7 is required for efferocytosis [9,22,23], the following model of ABCA7 regulation in regular and inflammatory homeostasis emerges: ABCA7 performs key functions in regular homeostasis in neural immune cells supporting the cells’ vigorousness, is down-regulated when the cells assume an inflammatory phenotype and is brought back again to a high expression level to ensure a robust transition to a resolving phenotype during inflammation resolution. Chronic unresolving inflammation may keep ABCA7 levels permanently down and promote AD pathogenesis. Indeed, individuals with multiple sclerosis, a disease characterized by constantly high levels of pro-inflammatory cytokines in the brain, are predisposed to developing AD [53]. Future work will test this model of the role of ABCA7 in regular homeostasis, inflammation and AD pathogenesis.

## 5. Conclusions

ABCA7 belongs to regular homeostasis in human microglia, astrocyte and neuronal cells, is down-regulated by cholesterol depletion in microglia and astrocytes and is incompatible with IL-1β and TNFα inflammatory responses in microglia. ABCA7 does not respond to cholesterol depletion in human THP-1 macrophages, which is different from murine macrophages where cholesterol depletion has been demonstrated to increase ABCA7 levels. ABCA7 functions are also incompatible with the PMA-induced inflammatory response in human THP-1 cells. These findings are consistent with the hypothesis that ABCA7 maintains lipidostasis in the brain by removing a potentially neurodegenerative lipid from neural cells that arises in these cells during normal physiological activity. The new findings further suggest that the loss of ABCA7 during AD pathogenesis could occur either because of an onset of inflammation or a sudden change in cholesterol metabolism.

## Figures and Tables

**Figure A1:**
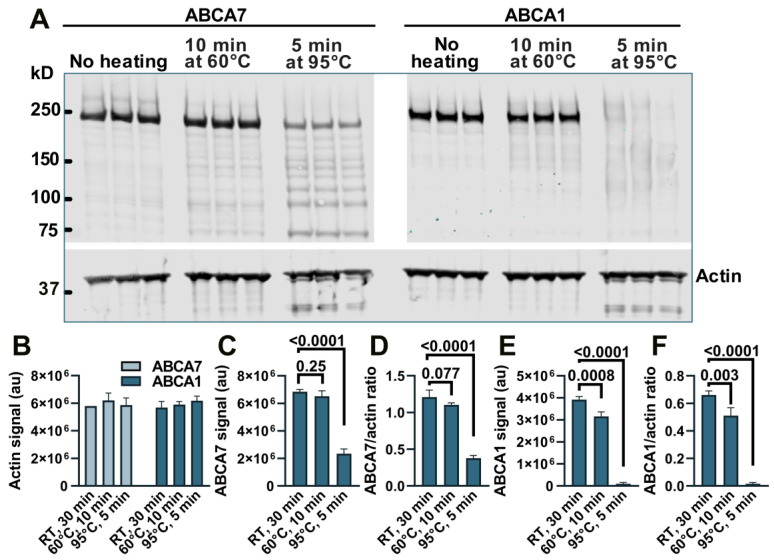
Heating causes a reduction in immunodetectable ABCA7 and ABCA1 in BHK-ABCA7 and BHK-ABCA1 cell lysate samples. (**A**) Samples for Western immunoblotting (aliquots of BHK-ABCA7 or BHK-ABCA1 cell lysates normalized to the same protein content, sample buffer, and reducing agent) were heat-treated as indicated, resolved on a polyacrylamide gel and transferred onto a nitrocellulose membrane. The membrane was cut horizontally at the 50 kDa marker, and then the upper portion was cut vertically at an empty lane between the BHK-ABCA7 and BHK-ABCA1 lysate sample lanes; each portion was probed with an appropriate primary antibody and an appropriate fluorescent dye-labeled secondary antibody. Quantification of fluorescence from the actin, ABCA7 and ABCA1 bands revealed that the heat treatment had no effect on the amount of detectable actin (**B**) but negatively affected the amounts of detectable ABCA7 (**C**,**D**) and ABCA1 (**E**,**F**). Detectable actin was quantified in fluorescence arbitrary units (au); detectable ABCA7 and ABCA1 were quantified in fluorescence au (**C**,**E**) and ratios of ABCA7 or ABCA1 fluorescence au to actin fluorescence au (**D**,**F**). Statistical analysis—ordinary ANOVA. RT, room temperature.

**Figure A2:**
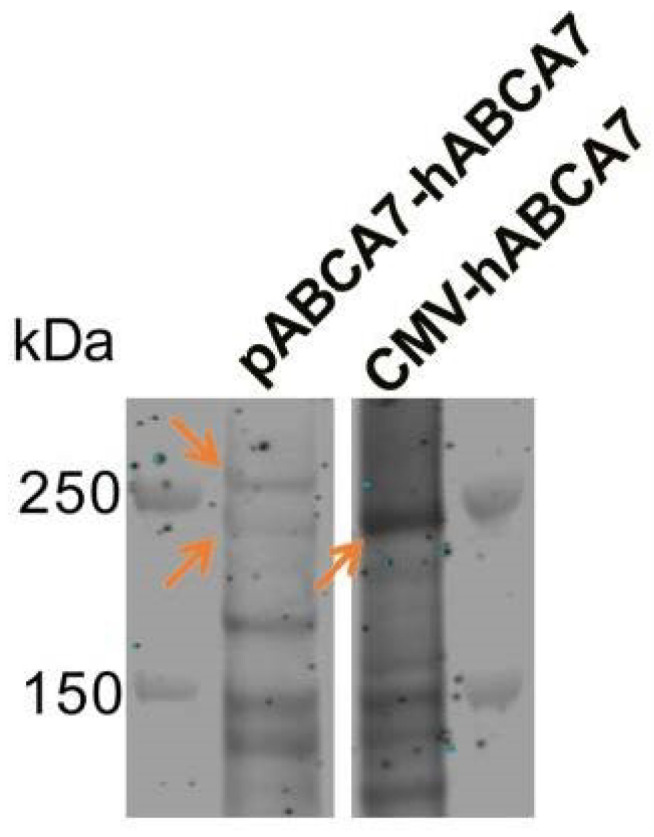
Expression of two ABCA7 isoforms (orange arrows in the left lane) when a construct (pABCA7-hABCA7) composed of the complete human *ABCA7* promoter, the first 11 exons, and a cDNA for the remaining *ABCA7* exons was expressed in BHK cells. The smaller, <250 kDa isoform migrated at the same molecular weight as the protein encoded in ENST00000263094.11 *ABCA7* mRNA expressed using the CMV promoter (orange arrow in the CMV-hABCA7 lane).

**Figure A3:**
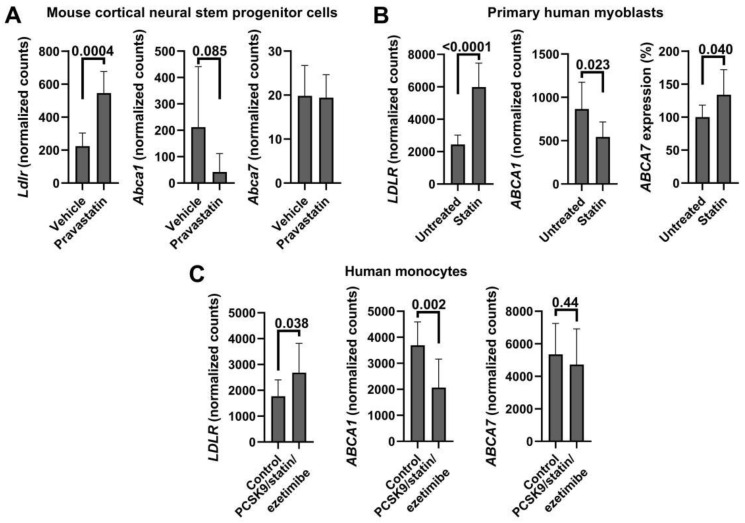
*ABCA7*/*Abca7* expression in cholesterol-depleted tissue in three publicly available RNA sequencing datasets. Normalized counts for *LDLR*/*Ldlr*, *ABCA1*/*Abca1* and *ABCA7*/*Abca7* were extracted and analyzed by unpaired *t*-test. Expression levels of *LDLR*/*Ldlr* and *ABCA1*/*Abca1* were significantly, correspondingly, increased and decreased in mouse cortical neural stem progenitor cells (**A**, [20]), primary human myoblasts (**B**, [58]) and human monocytes (**C**, [59]), but expression of *ABCA7*/*Abca7* significantly changed only in myoblasts. Ref. [58] datasets had normalized counts for untreated cells and cells treated with DMSO, rosuvastatin or simvastatin. To gain greater power, counts for untreated and DMSO-treated cells were combined and analyzed against the combined counts of rosuvastatin- and simvastatin-treated cells.

**Figure A4:**
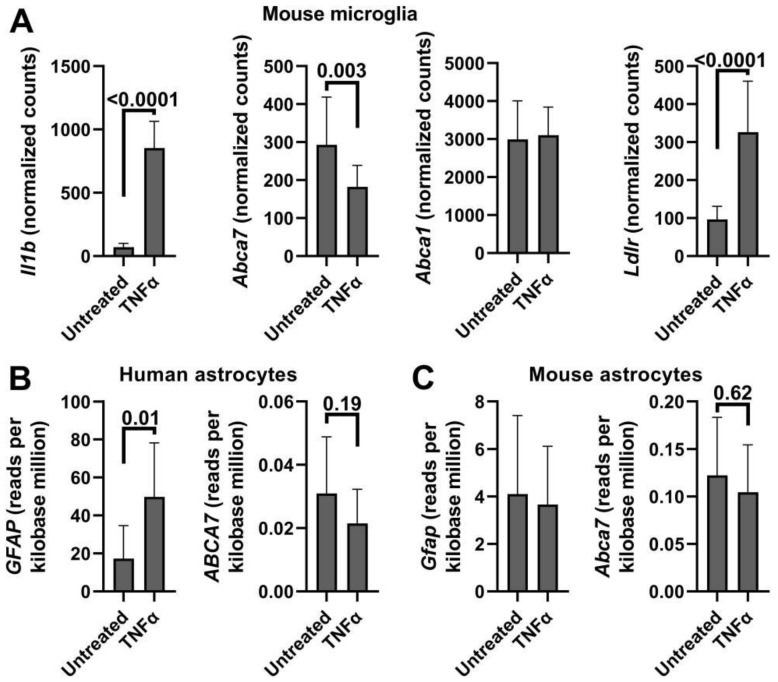
*ABCA7*/*Abca7* expression in TNFα-treated microglia and astrocytes in public datasets. Normalized counts or reads per kilobase million were extracted from the datasets, tested for normality and analyzed using the *t*-test. (**A**) These data are from ref. [48]. Expression of *Il1b* in this TNFα-treated microglia dataset was evaluated to determine whether the treatment was effective. (**B**,**C**) These datasets are from ref. [63].

## Data Availability

The data supporting the presented findings are contained within this article as numerical or graphical summary statistics. Raw data are available from the corresponding author. The GEO accession numbers of the public datasets used in this study are listed in Table A1 and Table A2.

## Appendix A. Loss of Immunodetectable ABCA7 and ABCA1 during Heating of Cell Lysates

We have previously noticed a major loss of immunodetectable ABCA7 when cell or tissue lysates were prepared for Western by heating at >90 °C and therefore heated samples at 60 °C [5,10], but a direct comparison of the heating temperatures with respect to the amount of ABCA7 detectable by Western in lysate samples has not been conducted. BHK-ABCA7 and BHK-ABCA1 cells were induced to express, correspondingly, ABCA7 and ABCA1 and lysed. Aliquots of the lysates (normalized to the same total protein amount) were combined with sample buffer and reducing agent and kept at room temperature, heated at 60 °C for 10 min, or heated at 95 °C for 5 min; the heat-treated samples were allowed to cool to room temperature, and all samples were resolved on a polyacrylamide gel (Figure A1A). The secondary antibodies used throughout this study were conjugated to a fluorescent dye. The heating had no effect on the amount of immunodetectable actin based on the fluorescence intensity of the actin bands in the BHK-ABCA7 and BHK-ABCA1 lysates (Figure A1B). The amounts of immunodetectable ABCA7 and ABCA1 were the highest in the samples kept at room temperature based on the fluorescence intensity of the ABCA7 and ABCA1 protein bands and the ABCA7/actin and ABCA1/actin fluorescence intensity ratios (Figure A1C–F). Heating at 60 °C had a negative but minor effect on the amount of immunodetectable ABCA7 (4.8 ± 5.79% and 8.7 ± 2.26% reductions based on, correspondingly, the ABCA7 band fluorescence and the ABCA7/actin fluorescence ratio) and a larger and significant effect on the amount of immunodetectable ABCA1 (19.4 ± 5.27% and 22.5 ± 8.70% reductions based on, correspondingly, the ABCA1 band fluorescence and the ABCA1/actin fluorescence ratio). Heating at 95 °C reduced immunodetectable ABCA7 by ~65% (based on either measure) and rendered ABCA1 nearly undetectable. In the present study, lysate samples in which ABCA7 was to be detected by Western were not heated above 60 °C, while lysates for ABCA1 detection were kept at room temperature.

## Appendix B. Existence of Two ABCA7 Isoforms

Strong co-expression of two ABCA7 isoforms has been reported for several cell types. Two isoforms of ABCA7 were expressed and regulated in the same manner in primary mouse macrophages [22]. Two isoforms, one migrating below and one migrating above the 250 kDa marker, were expressed and knocked down by the same *Abca7*-targeting siRNA in primary mouse brain capillary endothelial cells [54]. Two ABCA7 isoforms were expressed by human glioma cells (KNS-42), while human HeLa cells expressed the larger isoform and did not express a detectable level of the smaller isoform [55]. The mechanism that gives rise to the larger isoform is unclear at present. Differential splicing of the introns at the 5′ end of the *ABCA7* open reading frame has been previously reported [56]. Spliced human expressed sequence tags (ESTs) and mRNA transcripts in the NIH GenBank (e.g., ESTs DA193839 and DA119660 and mRNA AK310909) and the alternative splicing model for *ABCA7* derived by the transcriptome analyzer TromER (accession number NC_000019_55) indicate extensive alternative splicing in the first six introns and almost no alternative splicing in the remaining 40 introns of *ABCA7*. Indeed, two ABCA7 isoforms, one below and one above the 250 kDa marker, were detectable when we expressed the construct composed of the complete human *ABCA7* promoter, the first 11 exons, and a cDNA for the remaining *ABCA7* exons in BHK cells; the smaller <250 kDa isoform migrated at the same molecular weight as the protein encoded by the *ABCA7* mRNA ENST00000263094.11 expressed using the CMV promoter (Figure A2). However, none of the known human *ABCA7* ESTs or mRNAs encodes an isoform that would be large enough to migrate above the 250 kDa marker. In fact, if the sequence in introns 2–6 is expressed and is included in the ABCA7 protein (while ignoring stop codons), the resultant protein would not be large enough to migrate above the marker. Therefore, the larger isoform likely arises owing to post-translational modification.

When the protein encoded by ENST00000263094.11 was expressed in BHK cells, it was fully functional and mediated lipoprotein formation [10]. We have also previously shown that the <250 kDa isoform is by far the more predominant isoform in human brain lysates and induced pluripotent stem cells [5]. In human brain lysates, the <250 kDa isoform is associated with the progression of Alzheimer’s disease in 63–78-year-olds [5].

## Appendix C. Cholesterol Depletion and *ABCA7*/*Abca7* Expression in Public Datasets

To further evaluate the relationship between ABCA7 and cell cholesterol content, Google Scholar and GEO were searched for RNA sequencing and microarray datasets that contained results of cholesterol depletion experiments. Up-regulation of *LDLR*/*Ldlr* and down-regulation of *ABCA1*/*Abca1* expression in the identified datasets were taken as evidence of a successful decrease in cholesterol levels. Six datasets were deemed suitable (Table A1). Normalized counts available for three of these datasets were extracted and analyzed separately by *t*-test (Figure A3). Cholesterol depletion, as judged by a significant increase in *LDLR*/*Ldlr* and a significant decrease in *ABCA1*/*Abca1* expression, did not lead to a change in *ABCA7*/*Abca7* expression in human aortic smooth muscle cells, mouse liver, mouse cortical neural stem progenitor cells, human peripheral blood mononuclear cells or human monocytes (Table A1 and Figure A3A,C). However, ABCA7 was elevated by 33.9 ± 38.09% (*p* = 0.04, n = 8) in human cholesterol-depleted myoblasts (Figure A3B).

**Table A1:** Tissue, treatment and summary statistics for *LDLR*/*Ldlr*, *ABCA1*/*Abca1* and *ABCA7*/*Abca7* in the studies that investigated gene expression in cholesterol-depleted tissue.

Reference GEO Accession Number	Tissue Treatment (n)	Effect (Fold Change, *p*-Value) *		
		*LDLR*/*Ldlr*	*ABCA1*/*Abca1*	*ABCA7*/*Abca7*
Lu et al. [18] Not deposited	Human aortic smooth muscle cells 2-Hydroxypropyl-β-cyclodextrin 10 mg/mL vs. vehicle for 24 h (~53% reduction in cholesterol) (4 repeats)	**3.14** **0**	−**3.57** **0**	−0.12 0.80
Seo et al. [19] GSE28084	C57BL/6 mouse liver Lovastatin 100 mg and ezetimibe 21 mg per 100 g chow vs. powdered cholesterol 1% *w*/*w* of chow for one week (3 mice per group)	**1.42** **1.02 × 10^−7^**	−**0.28** **6.71 × 10^−4^**	0.024 0.67
Carson et al. [20] GSE111945	Mouse cortical neural stem progenitor cells 10 μ pravastatin vs. vehicle for 24 h (6 mice per group)	**2.4** **1.9 × 10^−7^**	**−5.0** **0.0079**	−1.02 0.89
Chamaria et al. [57] GSE86216	Human peripheral blood mononuclear cells Baseline vs. 8–12 weeks on 40 mg rosuvastatin daily (72 individuals)	**0.30** **5.41 × 10^−6^**	**−0.20** **1.59 × 10^−5^**	−0.028 0.42
Grunwald et al. [58] GSE107998	Primary human myoblasts 5 µM rosuvastatin vs. vehicle (4 cell populations)	**2.45** **9.25 × 10^−8^**	0.74 0.094	1.16 0.302
	5 µM simvastatin vs. vehicle (4 cell populations)	**2.39** **5.04 × 10^−7^**	**0.53** **0.008**	**1.5** **0.015**
Willemsen et al. [59] GSE192709 *	Human monocytes Patients with familial hypercholesterolemia treated with a combination of PCSK9 inhibitor, statin and ezetimibe vs. normolipidemic controls (10 per group)	N/A See Figure A3C		

* Fold change and *p*-values were obtained from GEO; summary statistics for Willemsen et al. [59] were not available. Fold change values and *p*-values in bold are statistically significant.

## Appendix D. *ABCA7*/*Abca7* Expression in IL-1β- or TNFα-Treated Microglia or Astroglia in Public Gene Expression Datasets

Google Scholar and GEO were searched for gene expression studies and datasets in which microglia or astroglia were treated with IL-1β or TNFα. A relatively large study (n = 9 and 11, [48]) was identified in which primary mouse microglia was subjected to TNFα (Table A2). The treated cells dramatically increased *Il1b* expression, indicating that application of TNFα was effective, and significantly down-regulated *Abca7* (Figure A4A). Notably in this microglia dataset, TNFα did not impact *Abca1* but significantly increased *Ldlr*, thus recapitulating the effect of cholesterol depletion (Figure A4A). Two studies were found in which mouse enteric glial cells, a type of astrocyte, or human fetal astrocytes were treated with IL-1β (Table A2). The treatment had no effect on *Abca7* in mouse enteric glia but reduced *ABCA7* in human astrocytes with a borderline level of statistical significance. A meta analysis on the *p*-values using the Fisher’s method gave the combined *p*-value of 0.13, indicating a lack of IL-1β effect on *Abca7* expression in astrocytes. Human or mouse primary astrocytes or neural stem cell-derived astrocytes were treated with TNFα in three studies and four datasets (Table A2; Figure A4B,C). The treatment had no effect on *Abca7* in the largest datasets (n = 9 and 6 for human donors and mice, respectively) but up-regulated the gene in the two smaller studies (n = 3 in each) with borderline statistical significance. A Fisher meta analysis on the *p*-values indicated that TNFα did not impact *ABCA7*/*Abca7* expression significantly in astrocytes (combined *p* = 0.32). There is no universal marker for reactive astrocytes; nonetheless, reactive astrocytes frequently but not always up-regulate expression of the gene for glial fibrillary acidic protein (GFAP) [60]. *GFAP*/*Gfap* expression was significantly increased in three of the six astrocyte datasets: one with reduced *ABCA7* expression owing to IL-1β treatment, one with no change in *ABCA7* after TNFα application and one with elevated *Abca7* after TNFα treatment (Table A2). The combined Fisher *p* = 0.31 for the three studies indicates a lack of association between the reactive state of astrocytes and *ABCA7* expression.

**Table A2:** Tissue, treatment and summary statistics for *ABCA7*/*Abca7* and astrocyte *GFAP*/*Gfap* in the studies that investigated gene expression in IL-1β- or TNFα-treated microglia or astroglia.

Reference GEO Accession Number	Tissue Treatment (n)	Effect (Fold Change, *p*-Value) *	
		*GFAP*/*Gfap*	*ABCA7*/*Abca7*
Delbridge et al. [48] GSE154853	Mouse microglia Untreated vs. 50 ng/mL TNFα for 24 h (n = 9 and 11)	Not applicable	**−0.38** **0.003**
Schneider et al. [61] GSE205610	Mouse enteric glial cells (a type of astrocyte) Untreated vs. 10 ng/mL IL-1β for 24 h (n = 3)	−1.1 0.87	−0.04 0.72
Lemaître et al. [62] GSE201555	Human fetal astrocytes Untreated vs. 20 ng/mL IL-1β for 48 h (n = 4 donors)	**1.1** **0.00001**	**−0.34** **0.041**
Li et al. [63] GSE147870	Human fetal astrocytes Untreated vs. 30 ng/mL TNFα for 48 h (n = 9 donors)	**1.9** **0.01**	−0.11 0.62
	Mouse astrocytes Untreated vs. 30 ng/mL TNFα for 48 h (n = 6)	−0.11 0.8	−0.29 0.35
Birck et al. [64] GSE117736	Neurospheres obtained from embryonic murine neural stem cells and then differentiated to astrocytes Untreated vs. 50 ng/mL TNFα for 24 h (n = 3)	**0.55** **0.013**	**0.24** **0.048**
Gabel et al. [65] GSE73022	Primary mouse astrocytes Untreated vs. 50 ng/mL TNFα for 24 h (n = 3)	0.51 0.13	**0.34** **0.037**

* Fold change and *p*-values were obtained from GEO or Figure A4. Fold change values and *p*-values in bold are statistically significant.
